# The Association Between Health Belief Model Components and Self-Care Practices Among Black/African American Men with Type 2 Diabetes

**DOI:** 10.3390/ijerph22030414

**Published:** 2025-03-12

**Authors:** Jeong-Hui Park, Ledric D. Sherman, Matthew Lee Smith, Megan S. Patterson, Tyler Prochnow

**Affiliations:** 1 Department of Health Behavior, School of Public Health, Texas A&M Health Science Center, 212 Adriance Lab Rd., College Station, TX 77843, USA; jeonghuipark@tamu.edu (J.-H.P.); lsherman@tamu.edu (L.D.S.); matthew.smith@tamu.edu (M.L.S.); megpatterson@tamu.edu (M.S.P.); 2 Center for Health Equity and Evaluation Research, Texas A&M University, College Station, TX 77843, USA; 3 Center for Community Health and Aging, Texas A&M University, College Station, TX 77843, USA

**Keywords:** health belief model, type 2 diabetes, self-care practice, Black/African American, men

## Abstract

This study investigates the impact of the Health Belief Model (HBM) constructs on self-care behaviors among Black/African American men with Type 2 Diabetes (T2D). A cross-sectional survey was conducted from February to June 2024, involving 1225 Black/African American men aged 21 years or older who self-reported a T2D diagnosis. The survey included HBM constructs, and self-care behaviors measured using established scales. Statistical analyses, including multiple linear regression, were performed to assess the relationships between HBM components and self-care practices, adjusted by demographic factors such as age, education, and income. The study found that self-efficacy was the strongest predictor of self-care behaviors across all domains (β = 0.055, *p* < 0.001). Perceived susceptibility was positively associated with healthful eating (β = 0.042, *p* = 0.038), glucose monitoring (β = 0.117, *p* < 0.001), and foot care (β = 0.140, *p* < 0.001). Perceived severity was positively linked to diet adherence (β = 0.015, *p* < 0.001) and glucose monitoring (β = 0.028, *p* < 0.001). Perceived barriers were negatively associated with self-care practices, including glucose monitoring (β = −0.047, *p* < 0.001). However, perceived benefits did not significantly impact overall self-care behaviors (β = 0.001, *p* = 0.893). Self-efficacy, perceived susceptibility, and perceived severity were significant predictors of self-care behaviors among Black/African American men with T2D. In particular, interventions aimed at enhancing self-efficacy, addressing barriers, and promoting awareness of diabetes risks may improve diabetes management and self-care behaviors in this population.

## 1. Introduction

Type 2 diabetes (T2D) is a chronic condition characterized by insulin resistance and an inability to produce sufficient insulin [1]. This leads to elevated blood glucose levels, which over time can cause serious health complications such as cardiovascular disease, nerve damage, kidney failure, and vision loss [2]. It is primarily influenced by lifestyle factors such as diet, physical inactivity, and obesity [1,3], although genetic predisposition also plays a significant role [4]. The global prevalence of T2D has been rising sharply, making it a critical public health issue. The prevalence of diagnosed diabetes in the U.S. is projected to increase dramatically, from 22.3 million (9.1% of the population) in 2014 to 39.7 million (13%) by 2030, and to 60.6 million (17%) by 2060 [5].

Structural inequities in social determinants of health fundamentally shape both T2D risk and management outcomes. These systemic factors include inequitable access to healthcare services, disparities in economic opportunities and resources, residential segregation affecting neighborhood environments, differential access to nutritious food and safe spaces for physical activity, and varying quality of educational and preventive health resources [6,7]. The compounded effects of these structural barriers create and perpetuate disparities in T2D prevention, diagnosis, treatment, and long-term management. Healthcare system experiences, including discrimination, provider bias, and institutional practices, further influence how individuals can engage with diabetes care and management [8]. Such inequities may place undue pressure or burden on individuals to self-manage their T2D in the absence of these support systems.

Further, these systemic inequities manifest in significant health disparities, particularly affecting Black/African American men who experience disproportionately higher T2D prevalence rates compared to the general population [9]. Beyond prevalence, Black/African American men face greater risks of severe complications including cardiovascular disease, kidney failure, and lower limb amputations [10]. Research demonstrates that these disparate outcomes may stem from structural barriers to preventive care, delays in diagnosis, limited access to diabetes education and support resources, and systemic obstacles to consistent care engagement [11]. Understanding how these men manage their T2D is essential for developing equitable approaches to support T2D self-management at all levels of health.

A crucial aspect of effective T2D management is the belief in the long-term benefits of T2D care, and this belief significantly influences patient adherence to management plans [12,13]. Patients who strongly believe in the benefits of their management are more likely to engage in regular physical activity, adhere to dietary recommendations, and consistently take their medications as prescribed [14]. These positive beliefs are associated with better glycemic control, fewer complications, and an improved quality of life [15]. For example, studies have shown that adults with T2D who have strong beliefs in their diabetes management plan exhibit better adherence to treatment protocols, leading to improved clinical outcomes [16,17]. Similarly, one previous study has demonstrated that adult patients with a positive outlook on their diabetes treatment experienced fewer complications and reported a higher quality of life [18]. Therefore, fostering beliefs of T2D care might be a powerful motivator for patients, leading to sustained engagement in healthy behaviors and adherence to treatment protocols.

Having positive beliefs about T2D self-care practices significantly influences lifestyle choices such as engaging in physical activity, alcohol consumption, and smoking habits [19,20]. Positive beliefs are associated with increased physical activity, which is crucial for managing blood glucose levels and reducing cardiovascular risks [21]. Moreover, strong beliefs in the benefits of diabetes care can lead to reduced alcohol consumption and smoking, behaviors that are known to exacerbate diabetes complications [22]. Studies have shown that individuals who maintain a healthy lifestyle, including limited alcohol intake and no smoking, have better health outcomes and a lower incidence of diabetes-related complications [23,24]. However, because most research on positive beliefs about T2D self-care practices focus on White/Caucasian or general populations, it remains unclear if Black/African American adults, especially men, experience the same outcomes. Research suggests that cultural, socio-economic, and healthcare access factors may shape health beliefs differently among Black/African American populations [25,26], potentially leading to distinct patterns in diabetes care and self-management practices compared to White populations. In addition, cultural beliefs and socio-economic disparities contribute to variations in self-care practices, with Black/African American adults often reporting more barriers to diabetes care compared to White individuals [27,28]. Therefore, it is crucial to focus on Black/African American adult men because they experience higher prevalence rates and more severe health outcomes with several challenges from T2D compared to other racial groups [29].

The Health Belief Model (HBM) comprises five key constructs that significantly influence health behaviors [30]: (1) *Perceived Susceptibility*, which reflects an individual’s belief in the likelihood of experiencing a health problem, thereby motivating preventive actions; (2) *Perceived Severity*, which pertains to the belief in the seriousness of the health issue and its potential consequences; (3) *Self-Efficacy*, which refers to an individual’s confidence in their ability to perform behaviors that can prevent or manage the disease; (4) *Perceived Benefits*, which highlights the positive outcomes that result from engaging in health-promoting behaviors; and (5) *Perceived Barriers*, which encompass the perceived obstacles that hinder the adoption of such behaviors [31]. The HBM is also a valuable theoretical framework for understanding how beliefs in the long-term benefits of diabetes care influence health behaviors, mental health, and adherence to monitoring practices among Black/African American men with T2D. The HBM indicates that health behaviors are a function of an individual’s perceptions of susceptibility to a health problem, the severity of the health problem, the benefits of avoiding the health problem, and the barriers to taking action [32]. Research has shown that perceived benefits and self-efficacy are crucial in motivating individuals to adhere to diabetes management plans, which can lead to better health outcomes [30,33]. For example, individuals who believe strongly in the effectiveness of their diabetes care plan are more likely to engage in regular physical activity, adhere to dietary recommendations, and consistently monitor their blood glucose levels [34]. Moreover, these positive beliefs can mitigate diabetes-related distress, anxiety, and depression, contributing to improved mental health and quality of life [35].

The primary objective of this study was to investigate the effects of the HBM constructs (e.g., perceived susceptibility, perceived severity, self-efficacy, perceived benefits, and perceived barriers) on self-care practices among Black/African American men with T2D. By emphasizing the role of health beliefs in T2D self-care, the current study seeks to understand how these constructs may improve adherence to treatment, health behaviors, and outcomes, particularly for Black/African American men with T2D.

## 2. Materials and Methods

### 2.1. Setting and Participants

The present study employed a cross-sectional design using data collected via a Qualtrics survey administered between February and June 2024. The internet-based survey aimed to assess attitudes and behaviors related to T2D among Black/African American adult men with T2D. A sample was obtained by recruiting and enrolling participants identified through Cloud Research. Potential participants were directed to an internet-based Qualtrics survey link and provided with an Institutional Review Board-approved information sheet ([anonymous]). Participants were assured of their anonymity, and informed consent was obtained from all respondents before participation. Data were collected and stored in a manner that ensured confidentiality. Participation was entirely voluntary, and respondents were informed of their right to withdraw from the survey at any time. Of the 3965 potential respondents, 1225 participants met the inclusion criteria: (1) self-identification as Black or African American, (2) male gender, (3) age 21 years or older, (4) self-reported T2D medical diagnosis, and (5) residence in the United States. Three quality/attention checks were included in the survey to ensure data integrity and enhance response validity. Respondents had to pass all three validity checks to be included in the final sample. These attention check questions required participants to answer a specific response (e.g., “Mark 4–6 times for this item”) to help prevent against careless answers.

### 2.2. Measures

#### 2.2.1. Independent Variables—HBM Constructs

The Diabetes Care Profile (DCP) questionnaire was designed to align with the constructs of the HBM by Fitzgerald and colleagues [36]. Several studies have used the DCP questionnaire for measuring health beliefs, self-care behaviors, and the perceived barriers and benefits of diabetes management [37,38,39,40]. These studies have shown that the DCP is effective in assessing the relationship between patients’ beliefs about their condition and their adherence to recommended diabetes care practices, such as medication adherence, glucose monitoring, and lifestyle changes, ultimately contributing to improved diabetes management outcomes [41].

**Perceived Susceptibility.** Perceived susceptibility refers to an individual’s belief about the likelihood of experiencing a health problem [42]. The Diabetes Care Profile—Control Problems Scale (DCP-CPS) [41] was employed to evaluate the frequency of specific diabetes-related events. This scale includes four items that measure the occurrence of particular symptoms and episodes over defined periods. Participants responded on a 6-point scale: 0 = Don’t know, 1 = 0 times, 2 = 1–3 times, 3 = 4–6 times, 4 = 7–12 times, and 5 = More than 12 times. The items addressed were: the frequency of low blood sugar reactions with symptoms (sweating, weakness, anxiety, trembling, hunger, headache) in the last month, severe low blood sugar reactions requiring assistance in the last year, high blood sugar symptoms (thirst, dry mouth, and skin, increased sugar in urine, reduced appetite, nausea, fatigue) in the last month, and the presence of ketones in urine in the last month. The endorsed items were aggregated to calculate a composite score reflecting each participant’s perceived susceptibility to T2D care, with potential scores ranging from 0 to 20 (Cronbach’s α = 0.86 [36]). This scale was chosen because it directly measures the frequency of events that influence an individual’s perception of their vulnerability to diabetes-related issues. The DCP-CPS is a valid and reliable tool that offers specific answers into how often participants experience events related to T2D, making it a strong indicator of perceived susceptibility.

**Perceived Severity.** Perceived severity involves the belief in the seriousness of the health issue and its consequences [42]. The Diabetes Care Profile—Social and Personal Factors Scale (DCP-SPFS) [41] was used to assess participants’ perceptions of the impact of diabetes on their lives, consisting of 13 items. For example, participants responded to the following item: “How often has your diabetes kept you from doing your normal daily activities during the past year (e.g., couldn’t: go to work, work around the house, go to school, visit friends)?”. Items were measured on a 5-point Likert scale, where 1 = strongly disagree, 2 = disagree, 3 = neutral, 4 = agree, and 5 = strongly agree. The items were combined to compute a composite score, with possible values ranging from 13 to 65 (Cronbach’s α = 0.85 to 0.90 [36]). This scale was chosen because it specifically measures how the perceived severity of diabetes affects individuals’ daily functioning, which is essential for understanding how severe diabetes is perceived to be by patients. By quantifying how diabetes affects participants’ daily lives, this scale provides an excellent reflection of the severity of the disease as understood by the individual.

**Self-Efficacy.** Self-efficacy refers to the confidence individuals have in their ability to successfully perform the actions required to prevent or manage a health condition [42]. Self-efficacy for T2D was assessed using the Self-Efficacy for Diabetes (SED) Scale, a widely recognized instrument designed to evaluate diabetes-specific self-efficacy [43]. Initially developed and validated for the Diabetes Self-Management study, this 8-item scale utilizes a 10-point rating system, with lower scores indicating diminished self-efficacy and higher scores reflecting increased self-efficacy. The SED Scale demonstrated strong reliability, with internal consistency (Cronbach’s α = 0.85) and test–retest reliability (intraclass correlation coefficient = 0.80) [44].

**Perceived Benefits.** Perceived benefits are the individual’s belief in the effectiveness of the recommended health behaviors in reducing the risk or seriousness of the health problem [42]. The Diabetes Care Profile—Long-Term Care Benefits Scale (DCP-LTCBS) [41] was utilized to evaluate participants’ beliefs about optimal diabetes care. This scale employs a 5-point Likert scale, where 1 = strongly disagree, 2 = disagree, 3 = neutral, 4 = agree, and 5 = strongly agree. Participants responded to items such as: “Taking the best possible care of diabetes will delay or prevent: eye problems”, “Taking the best possible care of diabetes will delay or prevent: kidney problems”, “Taking the best possible care of diabetes will delay or prevent: foot problems”, “Taking the best possible care of diabetes will delay or prevent: hardening of the arteries”, and “Taking the best possible care of diabetes will delay or prevent: heart disease”. All items were aggregated to compute a composite score representing each participant’s perceived beliefs of T2D care, with possible scores ranging from 5 to 25. The DCP-LTCBS was selected because it is specifically designed to capture beliefs about the benefits of diabetes care in preventing long-term complications, a critical factor in motivating self-care behaviors. The scale’s focus on tangible benefits, such as preventing complications, aligns well with the Health Belief Model’s framework, which suggests that individuals are more likely to engage in health-promoting behaviors if they perceive significant benefits.

**Perceived Barriers.** Perceived barriers are the individual’s assessment of the obstacles that might prevent them from taking action [42]. The Diabetes Care Profile—Monitoring Barriers and Understanding Management Practice Scales (DCP-MBUMPS) [41] were utilized to evaluate the barriers to diabetes monitoring and the frequency of management practices among participants. This scale comprises several items rated on a 5-point Likert scale, where 1 = rarely, 2, 3 = sometimes, 4, and 5 = often. Participants were asked how often they did not test for sugar as often as instructed due to various reasons, including forgetting, disbelief in the utility of testing, inappropriate timing or location, dislike of the task, running out of test materials, cost, inconvenience, difficulty in reading test results, inability to perform the test independently, infrequent changes in levels, and discomfort from finger pricks. The specific items assessed were: “When you don’t test for sugar as often as you have been told, how often is it because: you forgot?”, “you don’t believe it is useful?”, “the time or place wasn’t right?”, “you don’t like to do it?”, “you ran out of test materials?”, “it costs too much?”, “it’s too much trouble?”, “it’s hard to read the test results?”, “you can’t do it by yourself?”, “your levels don’t change very often?”, and “it hurts to prick your finger?”. The DCP-MBUMPS was chosen because it comprehensively assesses the diverse barriers to diabetes management, which can influence an individual’s decision to engage in self-care behaviors. In addition, this was selected for its ability to capture both practical and psychological barriers to diabetes management, offering a detailed view of the factors that can impede effective diabetes control.

#### 2.2.2. Dependent Variables

**Self-Care Practices for T2D.** The present study utilized the Summary of Diabetes Self-Care Activities (SDSCA) questionnaire to assess adherence to self-care practices for T2D [45]. This instrument is widely regarded as a convenient tool for evaluating diabetes self-care practices over a recent time frame, typically spanning the previous week or month. The 10-item SDSCA examines key domains of self-care, including dietary habits (4 items; two on general diet and two on specific diet), exercise (2 items), glucose monitoring (2 items), and foot care (2 items), by evaluating the frequency and consistency of participation in these activities [45]. The questionnaire has demonstrated adequate reliability across diverse cultural contexts, with Cronbach’s alpha values for the dietary habits subscale ranging from 0.60 to 0.80, and for other subscales (exercise, glucose monitoring, foot care) typically ranging from 0.50 to 0.70, which are considered acceptable for this type of instrument [45,46,47,48,49,50,51]. Using an 8-point Likert scale (ranging from 0 to 7 days), respondents reported the frequency of each self-care activity over the past 7 days. We calculated the average number of days per week that participants engaged in self-care management for T2D.

Specific questions for self-care practice measured in this study as follows: (1) general diet questions were: “How many of the last seven days have you followed a healthful eating plan?” and “On average, over the past month, how many days per week have you followed your eating plan?” The “healthful eating plan” was defined as the frequency with which participants adhered to a nutrition plan aligned with established diabetes management guidelines, including a balanced intake of carbohydrates, fats, and sugars. (2) Specific diet questions were: “On how many of the last seven days did you eat five or more servings of fruits and vegetables?” and “On how many of the last seven days did you eat high fat foods such as red meat or full-fat dairy products?”. Measured (3) exercise questions were: “On how many of the last seven days did you participate in at least 30 min of physical activity? (Total minutes of continuous activity, including walking)” and “On how many of the last seven days did you participate in a specific exercise session (such as swimming, walking, biking) other than what you do around the house or as part of your work?”. Also, the (4) glucose monitoring questions assessed were: “On how many of the last seven days did you test your blood sugar?” and “On how many of the last seven days did you test your blood sugar the number of times recommended by your health care provider?”. Lastly, (5) foot care were measured by: “On how many of the last seven days did you check your feet?” and “On how many of the last seven days did you inspect the inside of your shoes?”.

#### 2.2.3. Demographic Variables

Demographic factors were self-reported using several measures, encompassing age, educational attainment, marital status, employment status, annual household income, rurality, chronic condition, height, and weight, which were used to calculate the Body Mass Index (BMI). These variables were operationalized as follows: (1) age (21 years or older); (2) educational attainment (categorized as less than high school, some college or 2-year degree, and 4-year degree or higher); (3) marital status (classified as married/partnered, never married, divorced/separated, or widowed); (4) employment status (classified as student, employed, unemployed, retired, or unable to work); (5) annual household income (reported primarily in $25,000 USD increments); (6) rurality (classified as rural, suburban, urban, or other); (7) chronic conditions (18 chronic physical and mental health conditions were identified, including asthma, emphysema, chronic respiratory or lung issues, arthritis, rheumatic disease, cancer or cancer survivorship, chronic pain, diabetes, cardiovascular disease, hypercholesterolemia, hypertension, kidney disease, cognitive impairments, obesity, osteoporosis, obstructive sleep apnea, schizophrenia or other psychotic disorders, stroke, thyroid disorders, urinary incontinence, and other unspecified chronic conditions), and (8) the BMI (calculated as self-reported weight (pounds) divided by self-reported height (inches) 2, multiplied by 703) [52].

### 2.3. Data Analysis

All statistical analyses in this study were conducted using the statistical software STATA 18 (Stata Corporation, College Station, TX, USA). To ensure the validity of the statistical analyses, the assumptions for linear regression were thoroughly examined. We found that no issues with multicollinearity, normality of residuals, and the substantial outliers in this study. However, the Breusch–Pagan test revealed a significant violation of normality (*P*s < 0.05). Given the large sample size (n = 1225), this violation is expected to have a minimal impact on the analysis [53]. Additionally, further assessments of normality for individual dependent variables showed that it revealed acceptable normality (*P*s > 0.05). Based on these checks, the linear regression analysis was subsequently conducted. Descriptive statistics were computed to summarize the characteristics of the participants with frequency, mean, and standard deviation. Multiple linear regression was used to explore the relationships between the HBM components (independent variables) and self-care practices (dependent variables) among Black/African American men with T2D. All statistical significance was identified at *p* < 0.05.

## 3. Results

### 3.1. Participants’ Characteristics

The sample consisted of 1225 participants, with an average age of 41.9 years (±14.5). The sample demonstrated diversity in demographic characteristics and health profiles. A substantial proportion of participants (34%) had attained at least a four-year degree, reflecting a significant level of educational attainment within the sample. The majority of participants were employed (78.2%), and the residential distribution predominantly consisted of urban (52.4%) and suburban (36.1%) areas, which was consistent with broader population patterns observed in similar studies. Health-wise, participants reported an average of 2.5 chronic conditions (±1.9), indicating a moderate level of health burden. The mean BMI was 31.0 (±9.2), categorizing the sample as overweight to obese, suggesting that a significant portion of the sample was at increased risk for related health conditions. The variation in household income (~$3.9K ± 2.2K) highlighted the economic diversity of the participants. See Table 1 for the sample characteristics.

### 3.2. Linear Regression Analysis

#### 3.2.1. Perceived Susceptibility

This study found that perceived susceptibility had a significant positive association with overall self-care practices (β = 0.067, *p* < 0.001). Specifically, there was a significant association between perceived susceptibility and healthful eating plan adherence (β = 0.042, *p* = 0.038), glucose monitoring (β = 0.117, *p* < 0.001), and foot care (β = 0.140, *p* < 0.001). However, no significant association was observed in overall diet self-care (β = 0.026, *p* = 0.073) and exercise (β = 0.020, *p* = 0.353) (Table 2).

#### 3.2.2. Perceived Severity

Perceived severity demonstrated a significant positive association with overall diet self-care (β = 0.015, *p* < 0.001), in particular, healthful eating plan adherence (β = 0.020, *p* < 0.001) and weekly eating plan adherence (β = 0.022, *p* < 0.001) in general diet habits (β = 0.021, *p* < 0.001). Also, perceived severity showed the positive association with glucose monitoring (β = 0.028, *p* < 0.001). However, no significant relationship was observed between exercise (β = 0.009, *p* = 0.095) and foot care (β = 0.006, *p* = 0.360) (Table 2).

#### 3.2.3. Self-Efficacy

Self-efficacy was consistently positively associated with all outcomes, including not only overall self-care practice (β = 0.055, *p* < 0.001) but also overall diet self-care (β = 0.051, *p* < 0.001), exercise (β = 0.059, *p* < 0.001), glucose monitoring (β = 0.052, *p* < 0.001), and foot care (β = 0.059, *p* < 0.001) (Table 2).

#### 3.2.4. Perceived Benefits

Perceived benefits did not show any significant association with any of the overall self-care practices (β = 0.001, *p* = 0.893). Perceived benefits were significant associated with glucose monitoring (β = 0.024, *p* = 0.047), but there was no impact on overall self-care (β = 0.001, *p* = 0.893), overall diet self-care (β = −0.003, *p* = 0.654), exercise (β = 0.004, *p* = 0.705), and foot care (β = −0.020, *p* = 0.093) (Table 2).

#### 3.2.5. Perceived Barriers

Perceived barriers generally had a negative association with self-care practices (β = −0.013, *p* = 0.002). Specifically, higher perceived barriers were associated with less general diet habits (β = 0.005, *p* = 0.411) and less frequent glucose monitoring (β = −0.047, *p* < 0.001). However, no significant relationship was found with specific diet habits (β = −0.007, *p* = 0.114), exercise (β = −0.002, *p* = 0.706), and foot care (β = −0.001, *p* = 0.925) (Table 2).

## 4. Discussion

The primary aim of this study was to assess the relationship between the HBM constructs (e.g., perceived susceptibility, perceived severity, self-efficacy, perceived benefits, and perceived barriers) and self-care practices among Black/African American men with T2D. Overall, this study supported the HBM, finding self-efficacy as the most important predictor of positive self-care behaviors, with perceived susceptibility and severity also playing significant roles. Conversely, perceived barriers were found to impede self-care practices, and perceived benefits did not significantly influence overall self-care behaviors.

### 4.1. Perceived Susceptibility

Our results indicated that perceived susceptibility was positively associated with self-care practices, including adherence to a healthful eating plan, glucose monitoring, and foot care. This finding is consistent with the HBM, which suggests that individuals who perceive themselves as susceptible to a health problem are more likely to engage in preventive behaviors [54]. Furthermore, perceived susceptibility has been shown to drive health behaviors in diabetes management in various studies [55,56,57]. In the case of Black/African American men, heightened awareness of the risks associated with T2D, such as cardiovascular disease and kidney failure, may motivate individuals to adopt healthier behaviors to mitigate these risks [32]. Moreover, research has shown that heightened perceptions of susceptibility can lead to increased health-protective actions, such as medication adherence and lifestyle changes [55]. Our study builds on these findings by showing that perceived susceptibility is an important predictor of several diabetes self-care behaviors among Black/African American men.

### 4.2. Perceived Severity

Perceived severity, or the belief in the seriousness of diabetes and its potential complications, was positively associated with diet adherence and glucose monitoring, reinforcing the importance of recognizing the serious nature of the disease. The HBM posits that individuals who perceive a disease as severe are more likely to take action to prevent it or mitigate its consequences [58]. Our findings align with previous research that shows perceived severity influences health behaviors, including diabetes self-management [59,60]. For instance, when individuals perceive the consequences of uncontrolled diabetes, such as blindness or amputation, as severe, they are more likely to adhere to prescribed dietary plans and monitoring practices [61]. However, in our study, perceived severity did not significantly predict exercise or foot care behaviors. This discrepancy may be due to contextual factors such as socio-economic stressors and limited access to resources, which can diminish the perceived severity of health risks in populations facing multiple structural inequalities [62].

### 4.3. Self-Efficacy

Self-efficacy, or the belief in one’s ability to perform diabetes management tasks successfully, was the most robust predictor of self-care behaviors. These findings are consistent with the extensive body of literature on self-efficacy and its role in chronic disease management [63,64]. Self-efficacy has been identified as a key factor influencing engagement in health-promoting behaviors such as medication adherence, healthy eating, and participation in exercise [35,65]. Our study demonstrates that Black/African American men with T2D who believe in their ability to manage their condition are more likely to engage in self-care behaviors such as glucose monitoring and exercise. The positive impact of self-efficacy on diabetes management has been well-documented, and our results suggest that interventions aimed at increasing self-efficacy should be prioritized to improve health outcomes in this population. These interventions might include diabetes self-management education programs and psychological support aimed at enhancing individuals’ confidence in their ability to manage their disease [64].

### 4.4. Perceived Benefits

Interestingly, perceived benefits, or the belief that self-care practices will result in positive outcomes, did not significantly influence self-care behaviors in our study. Although participants recognized the benefits of diabetes care, such as the prevention of complications like kidney and eye problems, these beliefs did not translate into improved self-care behaviors. This finding is in line with a prior study that found while individuals with T2D may understand the benefits of managing their condition, competing barriers often prevent them from acting on these beliefs [18]. Barriers such as financial constraints, time limitations, and the perceived inconvenience of self-care may overwhelm the perceived benefits of care, thus hindering behavior change [66]. Our study adds to this literature by demonstrating that despite recognizing the importance of diabetes management, the perceived benefits alone may not be sufficient to drive behavior change in Black/African American men with T2D.

### 4.5. Perceived Barriers

Perceived barriers were negatively associated with self-care practices, indicating that the presence of barriers such as financial difficulties, lack of time, and the perceived inconvenience of self-care tasks hindered engagement in diabetes management behaviors. This finding aligns with previous research that highlights the significant role of barriers in influencing self-care behaviors [67,68]. In particular, financial constraints and the inability to afford medications or medical appointments are common barriers to effective diabetes management [69,70,71]. Our results emphasize the importance of addressing these barriers in interventions designed to improve diabetes self-care. For example, interventions could focus on reducing financial barriers, improving access to healthcare resources, and making self-care tasks more convenient and manageable for individuals with T2D.

### 4.6. Strengths and Limitations

This study has several strengths that enhance its validity and relevance in the field of T2D management, particularly among Black/African American men. Notably, it utilizes a large sample of Black/African American men with T2D, thereby improving the generalizability of the findings and improving the previous studies of minority populations in diabetes research. The focus on these specific demographics highlights critical health disparities and provides a basis for developing targeted interventions aimed at improving health outcomes in the same population. Despite these strengths, the study also has several limitations that may affect the interpretation of the results. The cross-sectional design inherently limits the ability to draw causal conclusions regarding the relationships between the study variables. Additionally, the use of self-reported data introduces the possibility of recall bias, as participants may inaccurately report their behaviors and practices, either overestimating or underestimating their actions. Furthermore, most of the variables assessed in this study were derived from the DCP questionnaire, which is grounded in the HBM. However, it is important to note that some of the variables, while closely aligned with the HBM components, may not fully map onto all aspects of the model. Lastly, the lack of longitudinal data restricts the ability to track how components of the HBM evolve over time, particularly in response to mediations designed to examine self-care practices. Without this longitudinal perspective, the long-term impacts of these interventions on T2D management and health outcomes remain uncertain.

## 5. Implications

The findings of this study have important implications for future practice in diabetes management, particularly for Black/African American men with T2D. Given that self-efficacy and perceived susceptibility were identified as key predictors of positive self-care behaviors, interventions should focus on strategies that enhance individuals’ confidence in their ability to manage their condition among this population. Health programs should incorporate elements that build self-efficacy, such as diabetes self-management education, skills training, and psychological support. Furthermore, addressing perceived barriers, such as financial difficulties and access to healthcare resources, is crucial in improving diabetes care. Public health practitioners should work to reduce these barriers by advocating for policies that increase access to affordable care, medications, and support services. Tailored interventions, which take into account the unique cultural and social determinants like socio-economic challenges faced by Black/African American men, should be prioritized to ensure these individuals have the tools and resources necessary to engage in consistent and effective self-care practices. Future studies should focus on further exploring the impact of social determinants of health, such as socio-economic status, healthcare access, and community support, to develop strategies that improve diabetes self-care and reduce health disparities among Black/African American men.

## 6. Conclusions

This study highlighted the significant role of self-efficacy in promoting self-care practices among Black/African American men with T2D. Our findings underscored the importance of fostering self-efficacy to improve diabetes management behaviors, as individuals with higher self-efficacy are more likely to engage in self-care behaviors. The study also reveals that perceived susceptibility and perceived severity positively influence self-care practices, while perceived barriers hinder engagement in diabetes management. This suggests that enhancing self-efficacy, rather than focusing solely on the perception of health risks or barriers, may be a more effective approach to improving self-care practices and overall health outcomes. Future research, particularly longitudinal studies, is needed to explore the long-term impacts of T2D management. Overall, these findings can provide important implications for developing tailored interventions/program to address health disparities and promote better self-care practices among Black/African American men with T2D.

## Figures and Tables

**Table 1:** Participants’ characteristics.

Variables		Total (n = 1225) % or M (±SD)
**Age (year)**		41.9 (±14.5)
**Education Level**		
	High School or Less	23.1%
	Some High School/2-Year Degree/No degree	42.9%
	4-Year Degree or More	34.0%
**Marital Status**		
	Married/Partnered	61.1%
	Never Married	27.6%
	Divorced/Separated	8.8%
	Widowed	2.5%
**Employment**		
	A student	1.9%
	Employed	78.2%
	Disabled	4.4%
	Retired	9.7%
	Not Employed	5.9%
**Rurality**		
	Rural	11.1%
	Suburban	36.1%
	Urban	52.4%
	Other	0.3%
**Household Income (~$25K increments)**		3.9 (±2.2)
**Number of Chronic Conditions**		2.5 (±1.9)
**Body Mass Index (kg/m^2^)**		31.0 (±9.2)

Note. M: Mean; SD: Standard Deviation.

**Table 2:** Association between the Health Belief Model components and self-care practice in multiple linear regression.

Variables	Overall Self-Care Practice	Total Diet Self-Care	Healthful Eating Plan Adherence	Weekly Eating Plan Adherence	Fruit and Vegetable Consumption	High Fat Food Consumption	Total Exercise	Physical Activity Participation	Specific Exercise Participation	Total Glucose Monitoring	Blood Sugar Testing	Recommended Blood Sugar Testing	Total Foot Care	Foot Check	Shoe Inspection
	β	β	β	β	β	β	β	β	β	β	β	β	β	β	β
Perceived Susceptibility	0.067 ***	0.026	0.042 *	−0.002	0.021	0.041	0.020	−0.006	0.046	0.117 ***	0.104 ***	0.128 ***	0.140 ***	0.125 ***	0.155 ***
Perceived Severity	0.014 ***	0.015 ***	0.020 ***	0.022 ***	0.009	0.009	0.009	0.010	0.008	0.028 ***	0.031 ***	0.025 ***	0.006	−0.002	0.013
Self-Efficacy	0.055 ***	0.051 ***	0.065 ***	0.065 ***	0.060 ***	0.015 ***	0.059 ***	0.059 ***	0.058 ***	0.052 ***	0.049 ***	0.055 ***	0.059 ***	0.058 ***	0.060 ***
Perceived Benefits	0.001 ***	−0.003	0.008	0.003	−0.004	−0.021	0.004	0.014	−0.005	0.024 *	0.023	0.026 *	−0.020	0.006	−0.047 **
Perceived Barriers	−0.013 **	−0.007	−0.020 **	−0.017 **	−0.008	0.017 *	−0.002	−0.006	0.001	−0.047 ***	−0.045 ***	−0.048 ***	−0.001	−0.001	0.001

Note. * *p* < 0.05, ** *p* < 0.01, *** *p* < 0.001. The analysis was adjusted by demographic factors (e.g., age, education status, employment level, marital status, employment status, annual household income, rurality, chronic condition, and the body mass index).

## Data Availability

The datasets used and/or analyzed during the current study are available from the corresponding author on reasonable request.

## References

[1] Handy R.M., Holloway G.P. (2023). Insights into the development of insulin resistance: Unraveling the interaction of physical inactivity, lipid metabolism and mitochondrial biology. Front. Physiol..

[2] Camell C.D., Günther P., Lee A., Goldberg E.L., Spadaro O., Youm Y.-H., Bartke A., Hubbard G.B., Ikeno Y., Ruddle N.H. (2019). Aging induces an Nlrp3 inflammasome-dependent expansion of adipose B cells that impairs metabolic homeostasis. Cell Metab..

[3] Obermayer A., Tripolt N.J., Pferschy P.N., Kojzar H., Aziz F., Müller A., Schauer M., Oulhaj A., Aberer F., Sourij C. (2023). Efficacy and safety of intermittent fasting in people with insulin-treated type 2 diabetes (INTERFAST-2)—A randomized controlled trial. Diabetes Care.

[4] Das S.K., Elbein S.C. (2006). The genetic basis of type 2 diabetes. Cellscience.

[5] Powers M.A., Bardsley J.K., Cypress M., Funnell M.M., Harms D., Hess-Fischl A., Hooks B., Isaacs D., Mandel E.D., Maryniuk M.D. (2020). Diabetes self-management education and support in adults with type 2 diabetes: A consensus report of the American Diabetes Association, the Association of Diabetes Care & Education Specialists, the Academy of Nutrition and Dietetics, the American Academy of Family Physicians, the American Academy of PAs, the American Association of Nurse Practitioners, and the American Pharmacists Association. J. Am. Pharm. Assoc..

[6] Hill-Briggs F., Adler N.E., Berkowitz S.A., Chin M.H., Gary-Webb T.L., Navas-Acien A., Thornton P.L., Haire-Joshu D. (2020). Social Determinants of Health and Diabetes: A Scientific Review. Diabetes Care.

[7] Wang J., Geng L. (2019). Effects of Socioeconomic Status on Physical and Psychological Health: Lifestyle as a Mediator. Int. J. Environ. Res. Public Health.

[8] Williams D.R., Mohammed S.A. (2009). Discrimination and racial disparities in health: Evidence and needed research. J. Behav. Med..

[9] Beckles G.L. (2016). Disparities in the prevalence of diagnosed diabetes—United States, 1999–2002 and 2011–2014. MMWR Morb. Mortal. Wkly. Rep..

[10] Magliano D.J., Islam R.M., Barr E.L., Gregg E.W., Pavkov M.E., Harding J.L., Tabesh M., Koye D.N., Shaw J.E. (2019). Trends in incidence of total or type 2 diabetes: Systematic review. BMJ.

[11] Royster M.O., Richmond A., Eng E., Margolis L. (2006). Hey brother, how’s your health? A focus group analysis of the health and health-related concerns of African American men in a southern city in the United States. Men. Masculinities.

[12] Galindo R.J., Trujillo J.M., Wang C.C.L., McCoy R.G. (2023). Advances in the management of type 2 diabetes in adults. BMJ Med..

[13] Niño-de-Guzman Quispe E., Bracchiglione J., Ballester M., Groene O., Heijmans M., Martínez García L., Noordman J., Orrego C., Rocha C., Suñol R. (2023). Patients’ and informal caregivers’ perspectives on self-management interventions for type 2 diabetes mellitus outcomes: A mixed-methods overview of 14 years of reviews. Arch. Public Health.

[14] Pot G.K., Battjes-Fries M.C., Patijn O.N., van der Zijl N., Pijl H., Voshol P. (2020). Lifestyle medicine for type 2 diabetes: Practice-based evidence for long-term efficacy of a multicomponent lifestyle intervention (Reverse Diabetes2 Now). BMJ Nutr. Prev. Health.

[15] Glasgow R.E., Fisher L., Strycker L.A., Hessler D., Toobert D.J., King D.K., Jacobs T. (2014). Minimal intervention needed for change: Definition, use, and value for improving health and health research. Transl. Behav. Med..

[16] Bai Y.L., Chiou C.P., Chang Y.Y. (2009). Self-care behaviour and related factors in older people with Type 2 diabetes. J. Clin. Nurs..

[17] Fisher L., Hessler D., Glasgow R.E., Arean P.A., Masharani U., Naranjo D., Strycker L.A. (2013). REDEEM: A pragmatic trial to reduce diabetes distress. Diabetes Care.

[18] Polonsky W., Fisher L., Hessler D., Edelman S. (2014). What is so tough about self-monitoring of blood glucose? Perceived obstacles among patients with type 2 diabetes. Diabet. Med..

[19] Kyrou I., Tsigos C., Mavrogianni C., Cardon G., Van Stappen V., Latomme J., Kivelä J., Wikström K., Tsochev K., Nanasi A. (2020). Sociodemographic and lifestyle-related risk factors for identifying vulnerable groups for type 2 diabetes: A narrative review with emphasis on data from Europe. BMC Endocr. Disord..

[20] Syed I.U. (2020). Diet, physical activity, and emotional health: What works, what doesn’t, and why we need integrated solutions for total worker health. BMC Public. Health.

[21] Reiner M., Niermann C., Jekauc D., Woll A. (2013). Long-term health benefits of physical activity–a systematic review of longitudinal studies. BMC Public Health.

[22] Pipatpiboon N., Koonrungsesomboon N., Suriyawong W., Sripetchwandee J., Turale S. (2022). Perception of benefits and barriers associated with dementia prevention behaviors among people with diabetes. Nurs. Health Sci..

[23] Thompson S. (2019). Supporting patients to make lifestyle behaviour changes. Nurs. Stand..

[24] Schmidt S.K., Hemmestad L., MacDonald C.S., Langberg H., Valentiner L.S. (2020). Motivation and barriers to maintaining lifestyle changes in patients with type 2 diabetes after an intensive lifestyle intervention (the U-TURN trial): A longitudinal qualitative study. Int. J. Environ. Res. Public Health.

[25] Campbell J.A., Yan A., Walker R.E., Weinhardt L., Wang Y., Walker R.J., Egede L.E. (2021). Relative Contribution of Individual, Community, and Health System Factors on Glycemic Control Among Inner-City African Americans with Type 2 Diabetes. J. Racial Ethn. Health Disparities.

[26] Egede L.E., Campbell J.A., Walker R.J., Linde S. (2023). Structural Racism as an Upstream Social Determinant of Diabetes Outcomes: A Scoping Review. Diabetes Care.

[27] Luebbert R., Perez A. (2016). Barriers to Clinical Research Participation Among African Americans. J. Transcult. Nurs..

[28] Walker A.Q. (2018). A Qualitative Exploration of Experiences and Motivations for Diabetes Self-Management in African American Men Between the Ages of 40–85 with Type 2 Diabetes. Ph.D. Thesis.

[29] Hackl C.M., Lee W.C., Sallam H.S., Jneid H., Campbell K.M., Serag H. (2024). Racial Disparities in Selected Complications and Comorbidities among People with Type 2 Diabetes. Healthcare.

[30] Champion V.L., Skinner C.S. (2008). The health belief model. Health Behav. Health Educ. Theory Res. Pract..

[31] Alyafei A., Easton-Carr R. (2025). The Health Belief Model of Behavior Change. StatPearls.

[32] Glanz K., Rimer B.K., Viswanath K. (2015). Health Behavior: Theory, Research, and Practice.

[33] Janz N.K., Becker M.H. (1984). The health belief model: A decade later. Health Educ. Q..

[34] Walker R.J., Smalls B.L., Hernandez-Tejada M.A., Campbell J.A., Davis K.S., Egede L.E. (2012). Effect of diabetes fatalism on medication adherence and self-care behaviors in adults with diabetes. Gen. Hosp. Psychiatry.

[35] Chew B.-H., Shariff-Ghazali S., Fernandez A. (2014). Psychological aspects of diabetes care: Effecting behavioral change in patients. World J. Diabetes.

[36] Fitzgerald J.T., Davis W.K., Connell C.M., Hess G.E., Funnell M.M., Hiss R.G. (1996). Development and validation of the Diabetes Care Profile. Eval. Health Prof..

[37] Bakri H.M., Alahmadi F.M., Taiyeb A.M., Khairallah H.H., Andijani A.M., Yaghmour K.A. (2022). The effect of family support, knowledge, and socioeconomic status in controlling diabetes and its complications on the patient. Middle East. J. Fam. Med..

[38] Alrasasimah W.A., Alsabaani A. (2024). Predictors of Diabetes Self-Management Behaviour Among Type 2 Diabetics in Saudi Arabia: A Cross-Sectional Study. Diabetes Metab. Syndr. Obes..

[39] Leong C.M., Lee T.-I., Chien Y.-M., Kuo L.-N., Kuo Y.-F., Chen H.-Y. (2022). Social media–delivered patient education to enhance self-management and attitudes of patients with type 2 diabetes during the COVID-19 pandemic: Randomized controlled trial. J. Med. Internet Res..

[40] Diriba D.C., Leung D.Y., Suen L.K. (2023). Effects of family-based diabetes self-management education and support programme on support behaviour amongst adults with type 2 diabetes in Western Ethiopia. Sci. Rep..

[41] Fitzgerald J.T., Anderson R.M., Gruppen L.D., Davis W.K., Aman L.C., Jacober S.J., Grunberger G. (1998). The reliability of the diabetes care profile for African Americans. Eval. Health Prof..

[42] Rosenstock I.M., Strecher V.J., Becker M.H. (1988). Social learning theory and the health belief model. Health Educ. Q..

[43] Stanford Patient Education Research Center (1990). Chronic disease self-management program questionnaire code book. Am. J. Public Health.

[44] Lorig K. (1996). Outcome Measures for Health Education and Other Health Care Interventions.

[45] Toobert D.J., Hampson S.E., Glasgow R.E. (2000). The summary of diabetes self-care activities measure: Results from 7 studies and a revised scale. Diabetes Care.

[46] Vincent D., McEwen M.M., Pasvogel A. (2008). The validity and reliability of a Spanish version of the summary of diabetes self-care activities questionnaire. Nurs. Res..

[47] Kamradt M., Bozorgmehr K., Krisam J., Freund T., Kiel M., Qreini M., Flum E., Berger S., Besier W., Szecsenyi J. (2014). Assessing self-management in patients with diabetes mellitus type 2 in Germany: Validation of a German version of the Summary of Diabetes Self-Care Activities measure (SDSCA-G). Health Qual. Life Outcomes.

[48] Ausili D., Bezze S., Canizzaro C., Bulgheroni M., Toolbert D.J., Genovese S., Di Mauro S. (2015). Self-care assessment in type-2 diabetes: The Italian translation and validation of the Summary of Diabetes Self-Care Activities. Prof. Inferm..

[49] Ansari R.M., Harris M.F., Hosseinzadeh H., Zwar N. (2021). Psychometric Evaluation and Validation of an Urdu Version of the Summary of Diabetes Self-Care Activities Measure (U-SDSCA). Am. J. Med. Qual..

[50] Adarmouch L., Sebbani M., Elyacoubi A., Amine M. (2016). Psychometric Properties of a Moroccan Version of the Summary of Diabetes Self-Care Activities Measure. J. Diabetes Res..

[51] Al Hashmi I., Al-Noumani H., Alaloul F., Murthi S., Khalaf A. (2022). Translation and psychometric validation of the Arabic version of Summary of the Diabetes Self-Care Activities (SDSCA) among pregnant women with gestational diabetes. BMC Pregnancy Childbirth.

[52] WHO (1995). Physical Status: The Use and Interpretation of Anthropometry: Report of a WHO Expert Committee.

[53] Field A. (2024). Discovering Statistics Using IBM SPSS Statistics.

[54] Abraham C., Sheeran P., Conner M., Norman P. (2015). The Health Belief Model.

[55] Yin J.D.-C., Lui J.N.-M. (2024). Factors influencing risk perception during Public Health Emergencies of International Concern (PHEIC): A scoping review. BMC Public Health.

[56] Taflinger S., Sattler S. (2024). A situational test of the health belief model: How perceived susceptibility mediates the effects of the environment on behavioral intentions. Soc. Sci. Med..

[57] Bekele N.T., Habtewold E.M., Deybasso H.A., Mekuria Negussie Y. (2024). Poor self-care practices and contributing factors among adults with type 2 diabetes in Adama, Ethiopia. Sci. Rep..

[58] Jones C.L., Jensen J.D., Scherr C.L., Brown N.R., Christy K., Weaver J. (2015). The Health Belief Model as an explanatory framework in communication research: Exploring parallel, serial, and moderated mediation. Health Commun..

[59] Hu Y., Liu H., Wu J., Fang G. (2022). Factors influencing self-care behaviours of patients with type 2 diabetes in China based on the health belief model: A cross-sectional study. BMJ Open.

[60] Daniel M., Messer L.C. (2002). Perceptions of disease severity and barriers to self-care predict glycemic control in Aboriginal persons with type 2 diabetes mellitus. Health Promot. Chronic Dis. Prev. Can..

[61] Perrier M.-J., Martin Ginis K.A. (2018). Changing health-promoting behaviours through narrative interventions: A systematic review. J. Health Psychol..

[62] Hill S.A. (2016). Inequality and African-American Health: How Racial Disparities Create Sickness.

[63] Daniali S.S., Darani F.M., Eslami A.A., Mazaheri M. (2017). Relationship between self-efficacy and physical activity, medication adherence in chronic disease patients. Adv. Biomed. Res..

[64] Slovinec D’Angelo M.E., Pelletier L.G., Reid R.D., Huta V. (2014). The roles of self-efficacy and motivation in the prediction of short-and long-term adherence to exercise among patients with coronary heart disease. Health Psychol..

[65] Komar-Samardzija M., Braun L.T., Keithley J.K., Quinn L.T. (2012). Factors associated with physical activity levels in African–American women with type 2 diabetes. J. Am. Assoc. Nurse Pract..

[66] Gholamnejad H., Darvishpoor Kakhki A., Ahmadi F., Rohani C. (2018). Barriers to self-care in elderly people with hypertension: A qualitative study. Work. Older People.

[67] Pun S.P., Coates V., Benzie I.F. (2009). Barriers to the self-care of type 2 diabetes from both patients’ and providers’ perspectives: Literature review. J. Nurs. Healthc. Chronic Illn..

[68] Mogre V., Johnson N.A., Tzelepis F., Hall A., Paul C. (2020). Barriers to self-care and their association with poor adherence to self-care behaviours in people with type 2 diabetes in Ghana: A cross sectional study. Obes. Med..

[69] Nam S., Chesla C., Stotts N.A., Kroon L., Janson S.L. (2011). Barriers to diabetes management: Patient and provider factors. Diabetes Res. Clin. Pract..

[70] Campbell D.J., Manns B.J., Hemmelgarn B.R., Sanmartin C., Edwards A., King-Shier K. (2017). Understanding financial barriers to care in patients with diabetes: An exploratory qualitative study. Diabetes Educ..

[71] Basu S., Garg S. (2017). The barriers and challenges toward addressing the social and cultural factors influencing diabetes self-management in Indian populations. J. Soc. Health Diabetes.

